# CircPIK3C2A Facilitates the Progression of Glioblastoma *via* Targeting miR-877-5p/FOXM1 Axis

**DOI:** 10.3389/fonc.2021.801776

**Published:** 2021-12-24

**Authors:** Jian Yang, Shuaiwei Tian, Baocheng Wang, Jiajia Wang, Liangliang Cao, Qinhua Wang, Wanqun Xie, Zhuangzhuang Liang, Heng Zhao, Yang Zhao, Keman Liao, Jie Ma

**Affiliations:** ^1^ Department of Pediatric Neurosurgery, Xinhua Hospital Affiliated to Shanghai Jiao Tong University School of Medicine, Shanghai, China; ^2^ Brain Injury Center, Renji Hospital, School of Medicine, Shanghai Jiao Tong University, Shanghai, China; ^3^ Department of Neurosurgery, Renji Hospital, School of Medicine, Shanghai Jiao Tong University, Shanghai, China

**Keywords:** CircPIK3C2A, glioblastoma, miR-877-5p, FOXM1, competing endogenous RNA (CeRNA)

## Abstract

Glioblastoma is a rare yet lethal type of tumor that poses a crucible for the medical profession, owing to its rapid proliferation and invasion resulting in poor prognosis. Circular RNAs (circRNAs), a subclass of regulatory RNAs, are implicated in the regulation of cancerous progression. This study aims to investigate the roles and underlying mechanism of circPIK3C2A in regulating proliferation and invasion of glioblastoma. qRT-PCR assays showed that the expression level of circPIK3C2A was aberrantly higher in glioblastoma cell lines, in comparison with that in normal glia cells. The ectopic expression of circPIK3C2A promoted the proliferation, invasion and clonal formation of glioblastoma cells, while circPIK3C2A loss-of-function exerted exactly the opposite biological effects on the cells. The construction of subcutaneous xenograft tumor model in nude mice indicated that circPIK3C2A loss-of-function effectively diminished tumor load *in vivo* and prolonged the survival time of tumor-bearing animals. Luciferase reporter assay confirmed the interaction among circPIK3C2A/miR-877-5p and *FOXM1*. CircPIK3C2A function as competitive endogenous RNA *via* sponging miR-877-5p through certain binding sites, thereby modulating the expression of FOXM1. Our results collectively indicate that circPIK3C2A functions as ceRNA by mediating miR-877-5p/FOXM1 axis, providing a novel perspective of applying CircPIK3C2A in the clinical intervention of glioblastoma in the future.

## Introduction

Glioblastoma multiforme (GBM) is the most common and aggressive type of brain cancer with grade IV histological malignancy, according to the classification of WHO (1). As a notoriously lethal type of central nervous system (CNS) tumor, GBM is characterized by swift cell proliferation, infiltrative migration, the induction of angiogenesis, rapid recurrence, and even radio-/chemoresistance (2). The patients bearing GBM has a median survival of merely 12 months, despite the intervention of traditional surgery, chemotherapy and radiation. Although significant advances have been made in the diagnosis and treatment of GBM, the clinical outcomes of patients receiving conventional treatments remains unsatisfactory. GBM patients may benefit from personalized therapies that targets certain molecular biomarkers, nonetheless, many potential treatments are counterbalanced by strong side effects in clinical trials or prove to be of little efficacy (3). Under such circumstances, it is necessary to perform intensified exploration into the molecular mechanism and the intricate gene regulation network that underlies the onset and progression of GBM, so as to determine novel therapeutic candidates and devise more promising treatment strategies for GBM.

Circular RNAs (circRNAs) represent a group of non-protein-coding RNAs characterized by the covalently closed loops without polyadenylation at their 5ʹ-caps and 3ʹ-tails (4). Predominantly distributed in the cytoplasm, CircRNAs are highly stable and can be well conserved across various species (4). Previous studies have claimed that circRNAs act as miRNA “sponges” by sharing common miRNA response elements(MREs) to regulate gene expression (5). circRNAs can bind to RNA-binding proteins (6) and can undergo translation (7). Even though plentiful circRNAs had been identified, not a few of them remain mysterious in tumorigenesis, not to mention the “sponges” of functional miRNAs in GBM. Zhang et al. reported the overexpression of circFOXO3 in GBM to promote GBM tumorigenesis (8). Consistently, Zheng et al. revealed that the deviant up-regulation of circTTBK2 in gliomas contributes to enhanced cell proliferation, migration and invasion (9). However, the exact biological function of circRNAs in GBM progression is largely unclear. More effort should be expended to reveal the role of circRNAs to advance the understanding of GBM pathogenesis, thereby contributing to the identification of new biomarkers or therapeutic targets of GBM. The biological function of circPIK3C2A(hsa_circ_0003577) has not been reported yet. We found that it is highly expressed in GBM cells, which remind us to investigate whether it play a crucial role in the tumorigenesis of GBM.

MicroRNAs (miRNAs) are small (18-25 nucleotides in length), non-coding endogenous RNAs that act as key regulators of gene expression. Numerous studies have demonstrated the dysregulation of miRNA expression in human cancers through various mechanisms (10, 11). miR-877-5p has been proved to a tumor suppressor that is involved in the development of several types of cancer, including hepatocellular carcinoma (HCC) (12), non-small cell lung cancer (NSCLC) (13), gastric cancer (14) and cervical cancer (15). miR-877-5p also functions to constrain the proliferative capacity of GBM cells (16). The specific mechanism that underlies the regulatory role of miR-877-5p in GBM advancement is yet to be clarified.

FOXM1, as a family member of Forkhead transcription factors, is involved in a myriad of cellular activities, including cell cycle progression (G1/S, G2/M transition), cell differentiation, DNA damage repair, cell proliferation, angiogenesis, apoptosis and tissue homeostasis (17, 18). As FOXM1 is over-expressed in a majority of human malignancies, it is also a target of regulation by multiple tumor suppressors. Moreover, Gong AH et al. demonstrated that FOXM1 overexpression promotes tumorigenicity of glioblastoma stem-like cells (GSC) (19). FOXM1 has emerged as a pivotal contributor to cancer development and progression, such as bladder cancer (20), breast cancer (21, 22), colorectal cancer (23), NSCLC (24, 25), pancreatic cancer (26), medulloblastoma (27), osteosarcoma (28), and hepatocellular carcinoma (29, 30). However, the potential role of FOXM1 in GBM invasiveness and its upstream regulatory mechanisms currently remain elusive.

In this study, we identified the expression pattern of circPIK3C2A in GBM cells, and then clarified the functional effect and underlying molecular mechanisms of circPIK3C2A in GBM progression through a series of *in vitro* and *in vivo* assays. Our data demonstrate that circPIK3C2A may function as an antagonist for GBM suppressor gene to facilitate the progression of GBM by mediating miR-877-5P/FOXM1 Axis. We hope that our research could contribute to the identification of potential therapeutic targets for the treatment of GBM.

## Materials and Methods

### Cell Culture

HEK293T, human GBM cell lines (U87-MG, U251-MG, A172 and T98G) and HEB (Normal brain glia cell line) were purchased from the Cell Bank of the Shanghai Branch of the Chinese Academy of Sciences. Cells were maintained in Dulbecco’s modified Eagle’s medium (DMEM) supplemented with 10% FBS in a 5% CO2 humidified atmosphere at 37°C.

### RNA Sample Treatment With RNase R and PCR

Total RNA was extracted from the treated cells by using Trizol (Thermo Fisher Scientific, USA), abiding by the instruction of the manufacturer. For RT-PCR assay, the treated RNA was directly reverse transcribed using Prime Script RT Master Mix (Takara, Japan). PCR was performed using PCR Master Mix (2x) (Thermo Fisher Scientific, Waltham, MA, USA). To quantify the amounts of circRNA and mRNA, real-time PCR analyses were performed using a SYBR Premix Ex TaqTM kit (Takara, Japan) by applying GAPDH as internal control. For miRNAs, reverse transcription of total RNA was performed using miScript II RT kit (QIAGEN, Germany) and miRNA expression levels were determined by miScript SYBR^®^ Green PCR Kit (QIAGEN, Germany), and U6 was used as the housekeeping control. The test of each sample was replicated three times and the data were analyzed by using the comparative CT method. The primers and RNA sequences are shown in **Supplementary Table 1**.

### Construction of Cells With Stable Knockdown or Overexpression of CircPIK3C2A

To construct circPIK3C2A overexpression (OE) plasmids, a basic sequence (flanked by HxoI and Agel) was synthesized. A small spacer sequence containing two restriction enzyme sites, HindIII and SalI, was added for the insertion of the circRNA fragment. GBM cells were seeded in six-well dishes at 2×10^5^ cells/well. On the following day, the cells were infected with virus at the same titer in the presence of 8 μg/mL polybrene. After 72 h of viral infection, the culture medium was replaced with selection medium containing 4 μg/mL puromycin. The cells were then cultured for at least 14 days. The puromycin-resistant cells were amplified in medium containing 2 μg/mL puromycin for seven to nine days and then transferred to medium without puromycin. Recombinant lentivirus and negative control (NC) lentivirus (Hanyin Co., Shanghai, China) were prepared and titred to 10^9^ TU (transfection unit)/mL. The KD or OE efficiency was confirmed by performing RT-qPCR assay.

### Fluorescence *In Situ* Hybridization Assays (FISH)

FISH assay was performed in U251 and T98G cells according to the manufacturer’s specifications using the Ribo™ Fluorescent In Situ Hybridization Kit. Cy3-labeled circPIK3C2A probes, U6 and 18S internal controls used in our study were designed and synthesized by RiboBio Co. Ltd. (Guangzhou, China). Briefly, cultured cells in 24-well plates were fixed with 4% paraformaldehyde at room temperature for 10 min. After permeabilization at 4°C for 5 min, the cells were incubated with specific probes at 37°C in the dark overnight. The cell nuclei was stained with 4,6-diamino-2-phenylindole (DAPI; 1 μg/ml, Thermo Fisher Scientific). Cells were observed and images were captured using a confocal microscope (Zeiss LSM 510 Meta, Germany).

### Cell Counting Kit-8, Colony Formation

After transfection, cells were seeded in 96-well plates at the density of 3000 cells/well for cell counting kit-8 (CCK-8) assays. 10 µl of CCK-8 reagent (Dojin, Japan) was added to each well, and the plate was incubated at 37°C for 1 h in dark. The OD was measured.

After transfection, cells were seeded in 6-well plates at 100 cells/well for colony formation assays. After 2 weeks of incubation, colonies (>200 cells per colony) were fixed with methanol and stained with crystal violet. The number of colonies was determined.

### Transwell Migration Assay

8 µm 24-well transwell chambers (Corning, USA) were used for migration assay. A total of 2×10^4^ GBM (U87-MG, U251-MG, A172, T98G) cells were seeded into the upper chambers and cultured with DMEM for 48 h at 37°C. Then, the cells on the upper membrane surface were removed by using a cotton swab. The cells on the lower surface of membrane surface were fixed using methanol and glacial acetic acid (ratio = 3:1) and the cells were stained with 10% Giemsa solution. Finally, 3 fields were selected randomly, and the number of migrated cells was counted for statistical analysis in each group.

### Matrigel Invasion Assay

Before seeding the cells, the poly-carbonate membranes of the transwell upper chambers (8 µm pore size; Corning, USA) was pre-coated with Matrigel (BD, USA). Then, 4×10^5^ cells, re-suspended in 200 µl serum-free medium, were placed into the upper chamber, followed by adding 600 µl of the same medium into the lower chamber. Then, the cells on the upper membrane surface were removed after 48 hours incubation at 37°C. Meanwhile, the cells on the lower membrane surface were fixed with methanol and glacial acetic acid (3:1). Subsequently, the cells were stained with 10% Giemsa solution. Finally, 3 fields were selected randomly and the cells in each field were counted for statistical analysis.

### Luciferase Reporter Assay

Plasmids were co-transfected with the predicted miRNAs or circRNAs into the cells by using Lipofectamine 3000 (Invitrogen, USA)-mediated gene transfer. The relative luciferase activity was determined as the ratio of firefly luciferase activity to Renilla luciferase activity at 48 h post-transfection. Each assay was carried out in three independent experiments.

### Western Blotting

Cell lysates were prepared with RIPA buffer containing protease inhibitors. Protein concentration was determined using a bicinchoninic acid protein assay kit. The immunoreactive bands were detected using an ECL kit (Pierce, PI32209; Thermo Fisher; USA). Primary antibodies targeting the following proteins were used: FOXM1 (cat#A2493; ABclonal,1:2000), and β-tubulin (cat#ab6046, Abcam; Cambridge; UK,1:5000). HRP-conjugated goat anti-rabbit/mouse IgG (0.2 µg/ml; Pierce31460 or 31430,1:200) were used as secondary antibodies. The chemical fluorescence images of proteins were visualized by using Luminescent Image Analyzer (Fujifilm, LAS-4000) after incubation with enhanced chemiluminescence reagents (Millipore, WBKLS0500).

### Animal Experiments

Nude mice were divided into three groups (n=8), including circPIK3C2A KD, circPIK3C2A NC, co-transfection of circPIK3C2A-KD and FOXM1 overexpressing. U87-MG cells were implanted into the corpus striatum of anesthetized athymic nude mice using a stereotaxic guidance. A sagittal incision was made through the skin to expose the cranium, and a burr hole was created in the skull at 0.2 mm anterior and 1.8 mm lateral from the bregma using a small dental drill. At a depth of 3 mm from the brain surface, 5 μL of cell suspension containing 1 ×10^6^ cells in PBS was injected into the brain. The needle was left in place for 5 min before retracting. Bone wax was used to seal the skull cavity, and the wound was sutured immediately. Tumor volumes were calculated as (length × width2)/2 using Function Analysis software (General Electric). The design and protocol of animal experiment were approved by the Institutional Animal Care and Use Committee of Shanghai Jiao Tong University.

### H&E Staining and Immunohistochemical (IHC) Staining

After 20 days of treatment in the intracranial orthotopic model, two mice from each group received ice-cold PBS and 4% formaldehyde *via* intracardiac perfusions. H&E staining for paraffin-embedded tissue sections was carried out by Servicebio biotech. Com (Wuhan, China). IHC was performed using the following primary antibodies: Ki67(cat#ab15580, Abcam; Cambridge; UK,1:50), cleaved caspase-3 (cat#9664; CST; M.A.; USA,1:50) and FOXM1 (cat#sc-376471; Santa Cruz Blotechnology,1:50).

### Statistical Analyses

All the data were presented in the form of mean ± standard deviation (SD) in the study. Two-tailed Student’s *t*-test was used to compare the differences between two groups, and log-rank test was used in the survival analysis. GraphPad Prism was used for data analysis and figure plotting. A *p* value of less than 0.05 was regarded as statistically significant.

## Results

### Expression of CircPIK3C2A in GBM Cells

**Figure 1:**
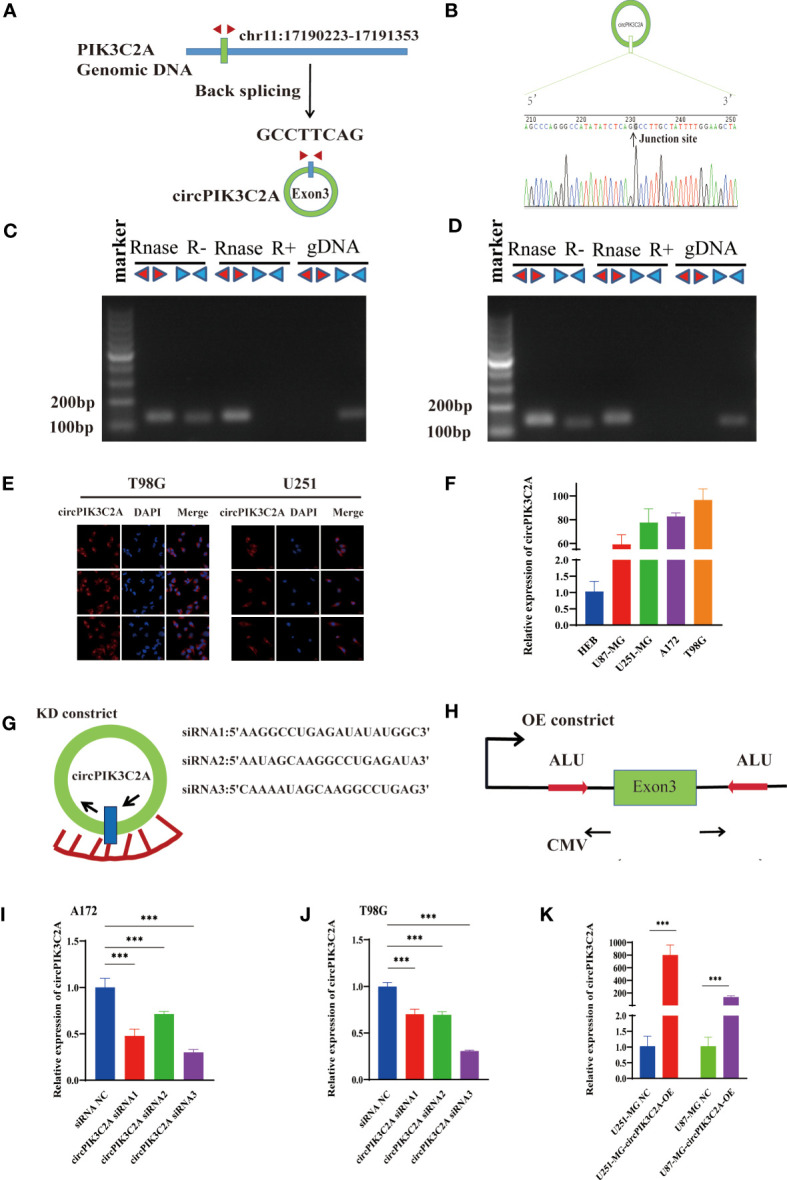
Identification and expression of circPIK3C2A in GBM cells. **(A)** The structure and part of the sequence of the junction of circPIK3C2A are provided, and divergent (red) primers were designed to amplify the back-splicing products. **(B)** Sanger sequencing after PCR using the indicated divergent flanking primers confirmed the “head-to-tail” splicing of circPIK3C2A. **(C, D)** The expression levels of the backspliced and canonical forms of PIK3C2A in cDNA and gDNA isolated from T98G and U251-MG cells. Red arrows represent divergent primers; blue arrows represent convergent primers. **(E)** CircPIK3C2A was detected by FISH in T98G and U251-MG. Nuclei were stained with 4′,6′-diamidino-2-phenylindole. Scale bar = 20μm. **(F)** Quantitative RT-PCR analysis of circPIK3C2A in GBM cells and HEBs. **(G)** A sketch of the short hairpin circPIK3C2A vector structure. **(H)** A sketch of the circPIK3C2A overexpression vector structure. ALU: Complementary ALU pairs were identified as at least one plus strand and one minus strand ALU element on opposite sides of the back-splice, which was required for the formation of circRNA; CMV: a common promoter in vectors. **(I–K)** Quantitative RT-PCR analysis of circPIK3C2A expression in GBM cells. Data are presented as the mean ± SEM of 3 independent experiments. Significant results are presented as ***<0.001.

CircPIK3C2A contains one exon that ultimately creates a transcript of 1310 nucleotides by back-splicing (**Figure 1A**). circPIK3C2A cDNA coincident with approximately 100 bp upstream and downstream from the junction site was amplified in GBM cells using divergent primers and was analyzed by using Sanger sequencing. The results confirmed the characteristics of circPIK3C2A junction (**Figure 1B**). The divergent primers detected circRNAs in cDNA with or without RNase R treatment, and the results showed that the circRNAs were truly circular and could not amplify any product from genomic DNA. The convergent primers amplified PCR products from linear PIK3C2A mRNA, yet these products vanished after RNase R treatment (**Figures 1C, D**). FISH analysis showed that circPIK3C2A was abundant and mostly located in the cytoplasm (**Figure 1E**). We determined circPIK3C2A expression levels in human normal glial cell (HEB) and 4 GBM cells (U87-MG, A172, U251-MG and T98G). Compared with that in HEB, circPIK3C2A was significantly upregulated in GBM cells (**Figure 1F**).

### CircPIK3C2A Promotes GBM Growth and Invasion *In Vitro*

**Figure 2:**
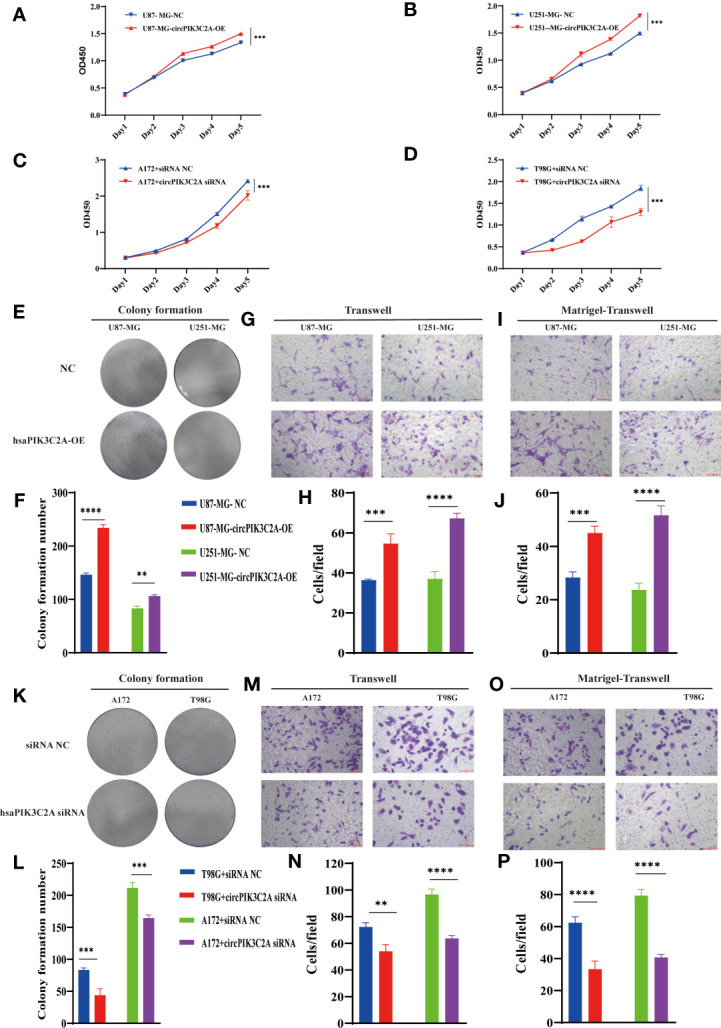
CircPIK3C2A affects GBM cell proliferation, colony formation, invasion, and migration. **(A–D)** CCK-8 assays were performed using U87-MG **(A)** and U251-MG **(B)** with or without circPIK3C2A- OE and using A172 **(C)** and T98G **(D)** cells with or without circPIK3C2A-KD. **(E–J)** Colony formation analyses **(E, F)**, transwell analyses **(G, H)** and matrigel-transwell assays **(I, J)** of U87-MG and U251-MG with or without circPIK3C2A-OE were performed. Representative staining images are presented (magnification: 200×; scale bar = 100 μm). **(K–P)** Colony formation analyses **(K, L)**, transwell analyses **(M, N)** and matrigel-transwel analyses **(O, P)** of A172 and T98G with or without circPIK3C2A-KD were performed. Representative staining images are presented (magnification: 200×; scale bar = 100 μm). Data are presented as the mean ± SEM of 3 independent experiments. Significant results are presented as **P < 0.01, ***P < 0.001, and ****P < 0.0001.

Then, to explore the function of circPIK3C2A, we overexpressed (circPIK3C2A-OE) or knocked down (circPIK3C2A-KD) circPIK3C2A in GBM cells. (**Figures 1G–K**). CCK-8 and colony formation assays showed that circPIK3C2A-OE enhanced the proliferative ability of U87-MG and U251-MG (**Figures 2A, B**). In addition, colony formation assays demonstrated that circPIK3C2A-OE increased the colony numbers (**Figures 2E, F**). Moreover, circPIK3C2A-KD inhibited the proliferation of A172 and T98G cells (**Figures 2C, D**), and significantly reduced the number of colonies (**Figures 2K, L**). We then evaluated the effects of circPIK3C2A on cell migration and invasion. As expected, circPIK3C2A overexpression promoted cell migration (**Figures 2G, H**) and invasion (**Figures 2I, J**), while the decreased expression of circPIK3C2A suppressed the migratory capability (**Figures 2M, N**) and invasive potential (**Figures 2O, P**) of cells. Taken together, these findings provide solid evidence that circPIK3C2A plays a crucial role of promoting GBM cells growth and invasion.

### CircPIK3C2A Serves as a Sponge of MiR-877-5p

**Figure 3:**
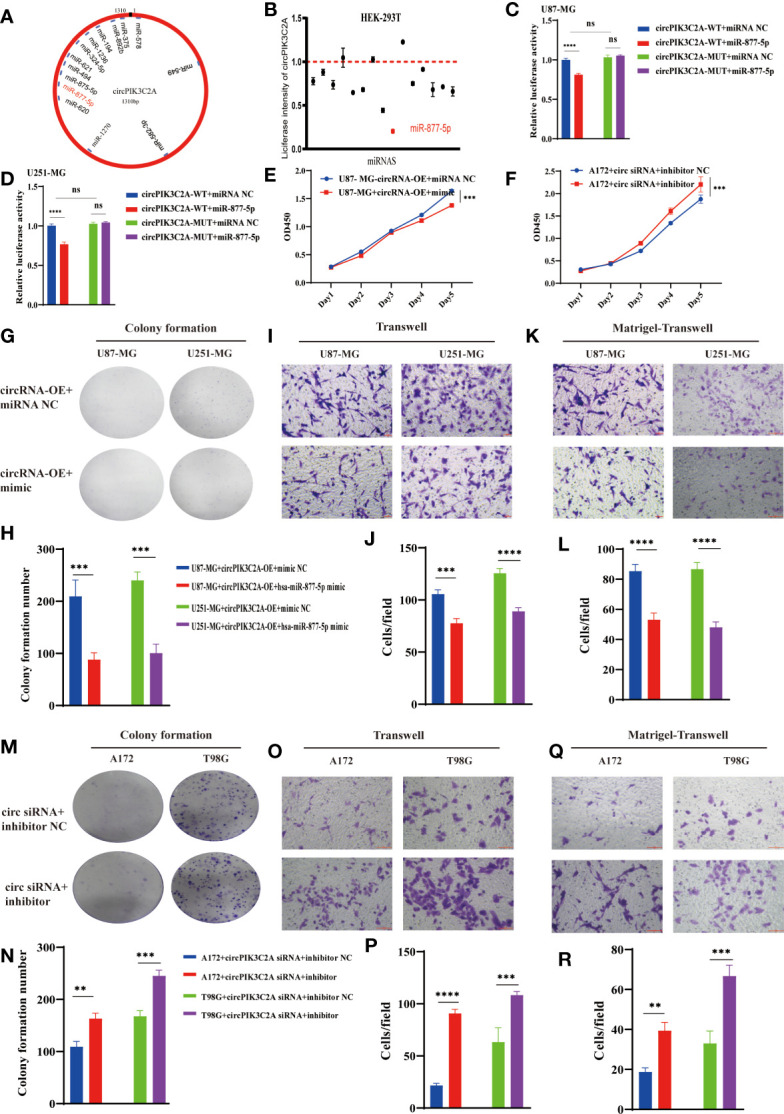
CircPIK3C2A function as a miR-877-5p sponge. **(A)** A schematic model shows the putative binding sites of 14 predicted miRNAs on circPIK3C2A. **(B)** Luciferase activity of circPIK3C2A in HEK293T cells transfected with miRNA mimics which are putative binding to the circPIK3C2A sequence. Luciferase activity was normalized by Renilla luciferase activity. **(C, D)** Luciferase activity assay was carried out to verify that miR-877-5p targets circPIK3C2A directly in GBM cells. **(E, F)** CCK-8 assays were performed after cells were co-transfected with different vectors. **(G–L)** Colony formation analyses **(G, H)**, transwell analyses **(I, G)** and matrigel-transwell analyses **(K, L)** of U87-MG and U251-MG with or without circPIK3C2A-OE+hsa-miR877-5p mimic were performed. Representative staining images are presented (magnification: 200×; scale bar = 100 μm). **(M–R)** Colony formation analyses **(M, N)**, transwell analyses **(O, P)** and matrigel-transwell analyses **(Q, R)** of A172 and T98G with or without circPIK3C2A siRNA+hsa-miR-877-5P inhibitor were performed. Representative staining images are presented (magnification: 200×; scale bar = 100 μm). Data are presented as the mean ± SEM of 3 independent experiments. Significant results are presented as^ ns^P < 0.05, **P < 0.01, ***P < 0.001, and ****P < 0.0001.

Considering the deep involvement of circPIK3C2A in GBM tumorigenesis and invasion, we then investigated the mechanisms underlying circPIK3C2A functions. Previous study has reported that circRNA regulates target gene expression by acting as ceRNAs for miRNAs in cytoplasm. FISH analysis revealed that circPIK3C2A was mainly distributed in the cytoplasm (**Figure 1E**). Fifty-eight candidate miRNAs were predicted to have binding sites along the circPIK3C2A sequence in the CircInteractome database, then 14 candidate miRNAs were selected above the 95th percentile of context+ score (**Figure 3A** and **Supplementary Table 2**). To validate the binding capability of these candidate miRNAs to circPIK3C2A, we established luciferase screening of a miRNA library. Each predicted miRNA mimics was co-transfected with circPIK3C2A luciferase reporter into HEK-293T cell. We found that multiple miRNAs were able to reduce luciferase activity and miR-877-5p reduced the most, to the extent of at least 25% (**Figure 3B**). The complementary base sequences between circPIK3C2A and miR-877-5p are shown in (**Supplementary Table 2**). Luciferase activity assay was conducted to verify the binding of circPIK3C2A to miR-877-5p. Our data showed that miR-877-5p reduced the luciferase activity level of the WT reporter gene of circPIK3C2A, whereas such effect could not be observed in the MUT reporter plasmid. Our data confirm that circPIK3C2A acts as an efficient “sponge” to bind miR-877-5p in GBM cells (**Figures 3C, D**). CCK-8 and colony formation assays were carried out to explore the effects of miR-877-5p on GBM cell proliferation. Our previous study shows that circPIK3C2A-OE enhances the proliferation, migration and invasion of U87-MG and U251-MG cells. Herein, we intend to ascertain whether miR-877-5p mimics could diminish these effects. The CCK-8 and colony formation assays showed that the proliferation ability of U87-MG and U251-MG cells was reduced co-transfected with circPIK3C2A-OE and miR-877-5p mimics compared with those in the circPIK3C2A-OE group (**Figures 3E, G, H** and **Supplementary Figure 1A**). In addition, compared with those in the circPIK3C2A-OE group, the cells co-transfected with circPIK3C2A-OE and miR-877-5p mimics exhibited compromised capacities of migration (**Figures 3I, J**) and invasion (**Figures 3K, L**) in U87-MG and U251-MG cells. Consistently, circPIK3C2A-KD could attenuate the downregulation of proliferation (**Figures 3F, M, N** and **Supplementary Figure 1B**) migration (**Figures 3O, P**) and invasion (**Figures 3Q, R**) promotion mediated by miR-877-5p in A172 and T98G cells.

### MiR-877-5p Suppress GBM Tumorigenesis and Invasion *In Vitro*

**Figure 4:**
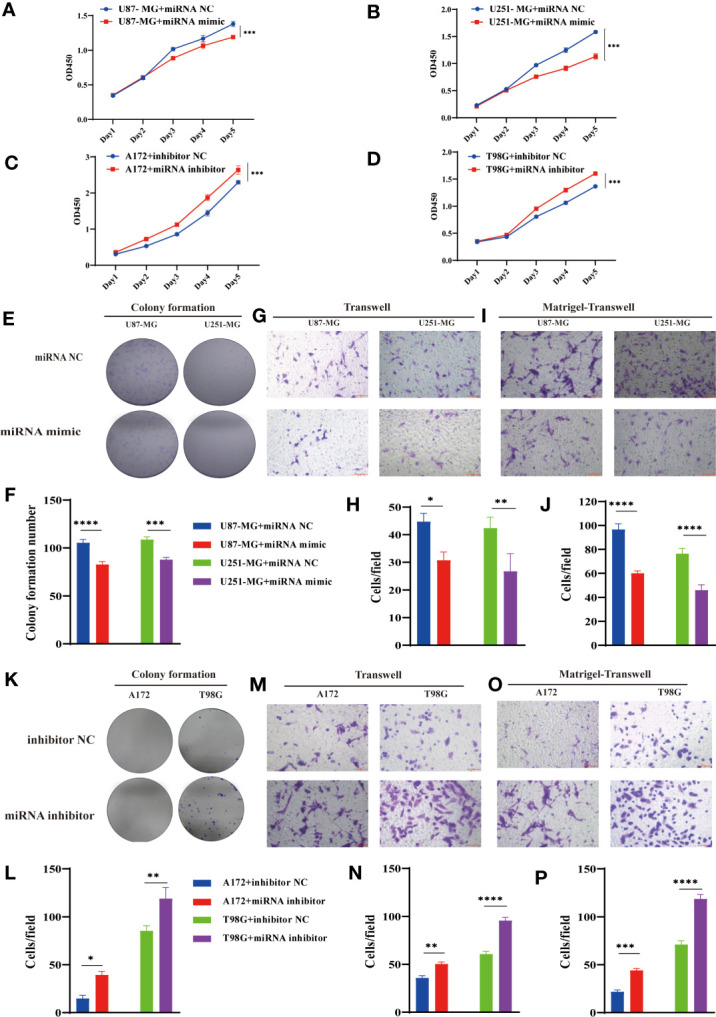
MiR-877-5p suppress cell proliferation, colony formation, invasion, and migration. **(A–D)** CCK-8 assays were performed using U87-MG **(A)** and U251-MG **(B)** with or without hsa-miR877-5p mimic and using A172 **(C)** and T98G(D) cells with or without hsa-miR877-5p inhibitor. **(E–J)** Colony formation analyses **(E, F)**, transwell analyses **(G, H)** and matrigel-transwell analyses **(I, J)** of U87-MG and U251-MG with or without hsa-miR877-5p mimic were performed. Representative staining images are presented (magnification: 200×; scale bar = 100 μm). **(K–P)** Colony formation analyses **(K, L)**, transwell analyses **(M, N)** and matrigel-transwell analyses **(O, P)** of A172 and T98G with or without hsa-miR877-5p inhibitor were performed. Representative staining images are presented (magnification: 200×; scale bar = 100 μm). Data are presented as the mean ± SEM of 3 independent experiments. Significant results are presented as *P < 0.05, **P < 0.01, ***P < 0.001, and ****P < 0.0001.

Considering the interaction between circPIK3C2A and miR-877-5p, we assessed the biological function of miR-877-5p in GBM. CCK-8 and colony formation assays revealed that overexpression of miR877-5p significantly reduced cell proliferative ability of the cancerous cells (**Figures 4A, B, E, F**). Moreover, functional inhibition of miR-877-5p promoted A172 and T98G cell proliferation (**Figures 4C, D**), significantly reduced the number of colonies (**Figures 4K, L**). Then, we evaluated the effects of miR-877-5p on cell migration and invasion by transwell assays. We found that miR-877-5p over-expression reduced cell migration (**Figures 4G, H**) and invasion (**Figures 4I, J**) while downregulation of miR-877-5p showed opposite effects (**Figures 4M–P**). These data indicated that miR-877-5p suppressed tumorigenesis and invasion in GBM cells.

### FOXM1 Is an Endogenous Target of MiR-877-5p

**Figure 5:**
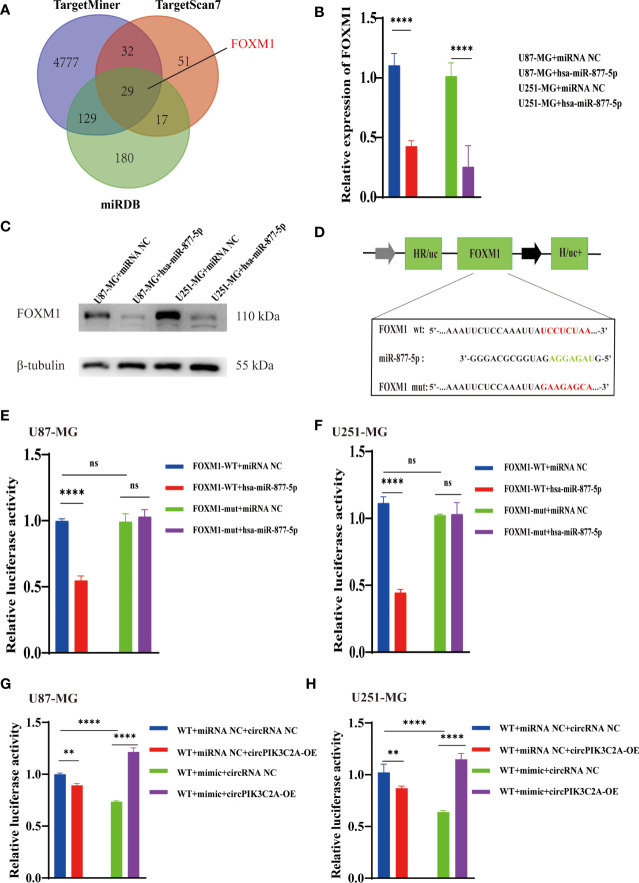
CircPIK3C2A modulates the expression of FOXM1, an endogenous target of miR-1877-5p. **(A)** Binding region of FOXM1 with miR-877-5p as predicted by bioinformatics analysis. **(B)** After transfection of miR-NC or miR-877-5p mimics into U87-MG and U251-MG cells, the expression level of FOXM1 was analyzed using qRT-PCR. **(C)** After transfection of miR-NC or miR-877-5p inhibitors into A172 and T98 cells, the expression level of FOXM1 was analyzed using qRT-PCR **(D)** A schematic drawing of the screening procedure for miR-877-5p candidate targets. **(E, F)** A luciferase reporter plasmid carrying wildtype (WT) or mutant (MUT) FOXM1 was co-transfected into U87-MG and U251-MG cells with miR-877-5p mimics in parallel with an empty vector. **(G, H)** A luciferase reporter plasmid carrying wildtype (WT) FOXM1 was co-transfected into U87-MG and U251-MG cells with or without miR-877-5p mimics or circPIK3C2A-OE. Significant results are presented as ^ns^P < 0.05, **P < 0.01, and ****P < 0.0001.

We predicted the targets of miR-877-5p and obtained 29 putative target genes by analyzing TargetMiner(http://www.isical.ac.in), miRDB(http://www.mirdb.org)and TargetScan7(http://www.targetscan.org/vert_71/) (**Supplementary Table 3**). Among these targets, we selected FOXM1, which has been reported to play a vital role in the occurrence and development of glioma (31, 32) as potential components of the circPIK3C2A-miR-877-5p ceRNA network (**Figure 5A**). the upregulation of miR-877-5p decreased FOXM1 expression (**Figure 5B**) Western blot analysis was conducted to evaluate the expression levels of FOXM1 following miR-877-5p overexpression (**Figure 5C**). The data indicated that miR-877-5p significantly inhibited the expression levels of FOXM1. To further elucidate the mechanism underlying the function of circPIK3C2A, the downstream targets of miR-877-5p were investigated. Luciferase activity assay was conducted to verify the binding of FOXM1 and miR-877-5p. The results showed that miR-877-5p decreased the luciferase activity of the WT reporter for FOXM1, but not that of the MUT-type reporter, which confirmed miR-877-5p as a sponge target of FOXM1 in U87-MG and U251-MG cells (**Figures 5D–F**). qPCR results indicated that miR-877-5p reduced the expression levels of FOXM1, while circPIK3C2A overexpression reversed the effects of miR-877-5p (**Figures 5G, H**). Our data reveal that circPIK3C2A functions as competing endogenous ceRNA to repress miR-877-5p, thereby mediating the expression of FOXM1.

### Inhibition of CircPIK3C2A Suppress the Growth of Xenografted Tumor *In Vivo*

**Figure 6:**
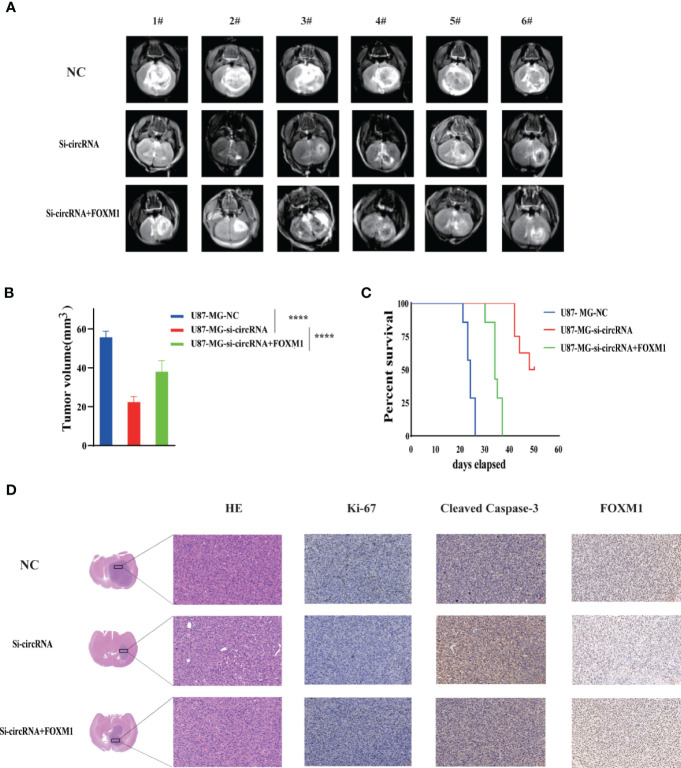
CircPIK3C2A KD inhibits GBM progression and FOXM1 overexpression reverses the effects of circPIK3C2A *in vivo.* **(A)** Representative MR images of xenograft GBM tumors orthotopically inoculated with cells with NC, circPIK3C2A- KD and circPIK3C2A-KD+FOXM1 on 3 weeks post-implantation. **(B)** Tumor volumes were calculated for each group (n = 8 per group). **(C)** The comparative survival of mice bearing NC, circFOXO3-KD and circPIK3C2A-KD+FOXM1 tumors was determined. The time of death was recorded as days after U87-MG implantation. **(D)** Representative hematoxylin and eosin images of each group are shown (magnification: 200×; scale bar =50μm). Representative immunohistochemistry images of Ki-67, cleaved caspase-3, FOXM1 in tumors collected from each group (magnification: 40×; scale bar = 50μm). Significant results are presented as ****P < 0.0001.

**Figure 7:**
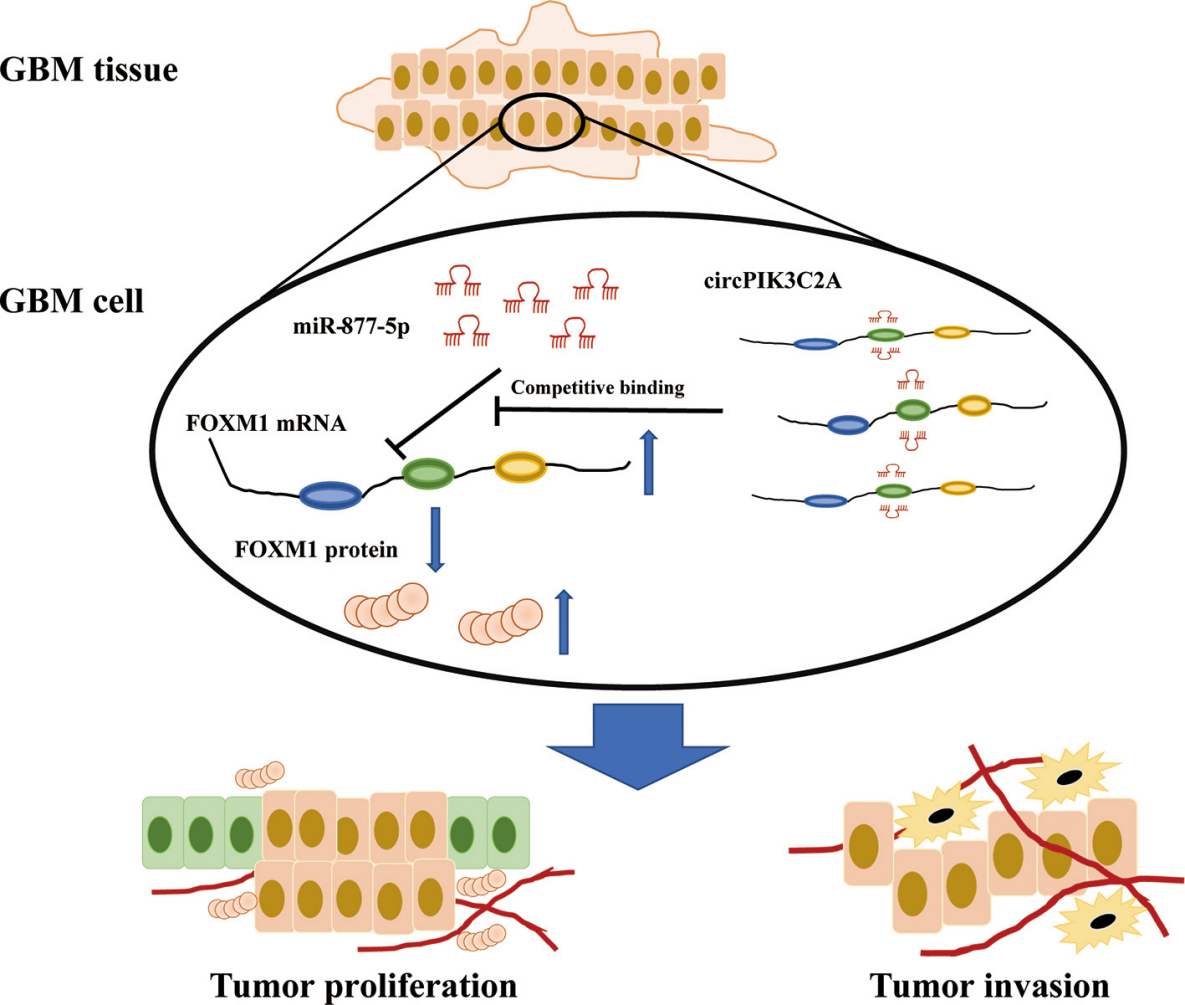
Schematic summary. In the current study, we provide novel insights into the mechanisms underlying circPIK3C2A promoting GBM progression and invasion. Mechanistically, circPIK3C2A sponges miR-877-5p, acts as a ceRNA to regulate FOXM1 expression and modulates tumor cell function *in vitro* and *in vivo*.

To determine the *in vivo* effect of circPIK3C2A on GBM progression, we injected circPIK3C2A-KD, circPIK3C2A-NC or circPIK3C2A-KD and FOXM1 overexpression (FOXM1-OE) con-transfected cells into male nude mice. The growth of tumors was monitored *via* MRI (**Figure 6A**), we found that circPIK3C2A-KD significantly reduced tumor growth and invasion, and FOXM1-OE effectively abolished the inhibitory effect of circPIK3C2A-KD as shown in hematoxylin-eosin (HE) and immunohistochemical (IHC) staining (Ki67+ for proliferating cells, cleaved caspase 3^+^ (CC3^+^) for apoptotic cells) results (**Figures 6A, D**). Tumor volumes were decreased by approximately 2-fold in circPIK3C2A-KD group, compared with that of circPIK3C2A-NC group. Tumor load in the animals bearing circPIK3C2A-KD and FOXM1 FOXM1-OE) con-transfected cells was higher than the circPIK3C2A-KD group (**Figure 6B**). Strikingly, the median survival of the mice implanted with circPIK3C2A-KD cells was prolonged to 48.6 days, while the mice implanted with co-transfection of circPIK3C2A-KD and FOXM1 overexpressing cells had a median survival of merely 34.2 days. All control animals died within 25 days (**Figure 6C**). These results reveal the critical role of circPIK3C2A in GBM progression and that FOXM1 overexpression could offset the biological function of circPIK3C2A-KD *in vivo*. Mechanistically, circPIK3C2A promotes the progression of GBM cells by sponging miR-877-5p and targeting *FOXM1* (**Figure 7**). Therefore, the disruption of circPIK3C2A/miR-877-5p/*FOXM1* axis could be a promising approach for the intervention of GBM.

## Discussion

Since the initial identification in 1976 by Sanger et al., circular RNAs were generally presumed as the accidental byproduct of aberrant RNA splicing. However, the in-depth investigations in recent years help the researchers to gain more insight into the several appealing features of circRNAs, such as high abundance, great stability, well-conserved sequence, as well as tissue-specific and stage-specific expression pattern. The various biological functions of circRNAs involve the regulation of miRNAs function, as circRNAs act as “sponge” to competitively bind to microRNAs, and thereby keeping their target genes away from microRNAs. CircRNAs that manifests deviant expression pattern have been reported to evoke the pathogenesis and progression of GBM (glioblastoma). CircRNAs have emerged as a research hotspot especially in the field of oncology.

To delve into the cellular and molecular mechanism underlying the pathogenesis of GBM comprehensively, we investigated the biological function of circPIK3C2A in GBM. We observed notably up-regulated circPIK3C2A level in GBM cell lines, as compared with that in HEB, and that circPIK3C2A knockdown inhibited the growth and invasion of GBM cells *in vivo* and *in vitro*. Intriguingly, our data showed that circPIK3CA mediates the expression of FOXM1 *via* competitively binding to miR-877-5p.

We analyzed the target microRNAs of circPIK3CA to explore the regulatory mechanism by applying bioinformatics software. Bioinformatics prediction confirmed that miR-877-5p was the target of circPIK3CA. We found the notably reduced level of miR-877-5p in tumor cells, a result consistent with the very notion that miR-877-5p being a tumor suppressor gene in a variety of tumors including glioma (31, 33, 34). Consistently, our subsequent experiment confirmed that miR-877-5p could specifically bind to the 3’-UTR region of *FOXM1*. The overexpression of circPIK3CA elevated the expression of FOXM1, whereas the up-regulation of miR-877-5p inhibited its expression. Noteworthily, inhibition of FOXM1 mediated by miR-877-5p gain-of-function could be rescued to a certain extent by the overexpression of circPIK3CA. The elevated mRNA expression of FOXM1 induced by miR-877-5p knockdown could be abolished in part by the up-regulation of circPIK3CA.

As a member of the forkhead-box family of transcription factor, *FOXM1* is an evolutionarily conserved gene that contains a DNA-binding domain known as forkhead-box domain. FOXM1 is a typical cell proliferation-related transcription factor that is implicated in mediating cell growth and cell cycle. Evidence suggested that FOXM1 is markedly up-regulated in GBM cells, and that the high FOXM1 expression level tends to indicate dismal prognosis for patients with GBM (28). Additionally, FOXM1 functions to promote glioma proliferation, stimulate epithelial-mesenchymal transition (EMT) (35, 36), strengthen the resistance of GBM cells to radiotherapy (37), and facilitate self-renewal and of glioma stem cells (GSCs) (19). Finally, *in vivo* assay of xenografted tumor in nude mice revealed that the overexpression of circPIK3CA resulted in inhibited growth of tumor cells while the inhibition of circPIK3CA markedly reduced the volume of solid tumor and thereby diminishing tumor load.

In the current study, we verify that *FOXM1* is a downstream target gene of miR-877-5p and is subject to the positive regulation of circPIK3CA in the tumor microenvironment (TME) of GBM. Our work reveals that cicrPIK3CA/miR-877-5p/*FOXM1* regulatory axis plays a crucial role in the progression of GBM and targeting circPIK3CA providing a therapeutic potential for GBM patients.

## Data Availability Statement

The original contributions presented in the study are included in the article/**Supplementary Material**. Further inquiries can be directed to the corresponding authors.

## Ethics Statement

The animal studies were approved by the Ethics Committee of Medical School, Shanghai Jiao Tong University (IRB number, B-2019-003).

## Author Contributions

JY, ST, JM, and KL conceived the project and designed all of the experiments. JY, ST, BW, JW, and YZ carried out most of the experiments. JY, JW, and KL, and JM wrote and revised the manuscript. Data analyses were performed by JY, JW, LC, ZL, WX, HZ, QW, and KL. All authors contributed to the article and approved the submitted version.

## Funding

This study received funding from the Shanghai Xin Hua Hospital (JZPI201701 to JM), Shanghai Shenkang Hospital Development Center (16CR2031B to JM), Shanghai Science and Technology Committee (17411951800 to JM), and Chinese National Science Foundation for Young Scholars (81702453 to YZ). Chinese National Science Foundation for Young Scholars (82002630 to KL) Shanghai Sailing Program (20YF1426200 to KL).

## Conflict of Interest

The authors declare that the research was conducted in the absence of any commercial or financial relationships that could be construed as a potential conflict of interest.

The reviewer WX declared a shared affiliation with the authors at the time of review.

## Publisher’s Note

All claims expressed in this article are solely those of the authors and do not necessarily represent those of their affiliated organizations, or those of the publisher, the editors and the reviewers. Any product that may be evaluated in this article, or claim that may be made by its manufacturer, is not guaranteed or endorsed by the publisher.

## Supplementary Material

Supplementary Figure 1 CCK-8 assays were performed after cells were cotransfected with different vectors. **(A)** U251-MG cells co-transfected with circPIK3C2A-OE and miR-877-5p mimics was reduced compared with those in the circPIK3C2A-OE group. **(B)** T98G cells co-transfected with circPIK3C2A-KD and miR-877-5p inhibitor was reduced compared with those in the circPIK3C2A-KD group.Data are presented as the mean ± SEM of 3 independent experiments. Significant results are presented as *P < 0.05, **P < 0.01, ***P < 0.001, and ****P < 0.0001.
