# Supplementary figures and images for: CircPIK3C2A Facilitates the Progression of Glioblastoma via Targeting miR-877-5p/FOXM1 Axis
Source: Front Oncol. 2021 Dec 24;11:801776. doi: 10.3389/fonc.2021.801776 (PMC8739489; doi:10.3389/fonc.2021.801776)

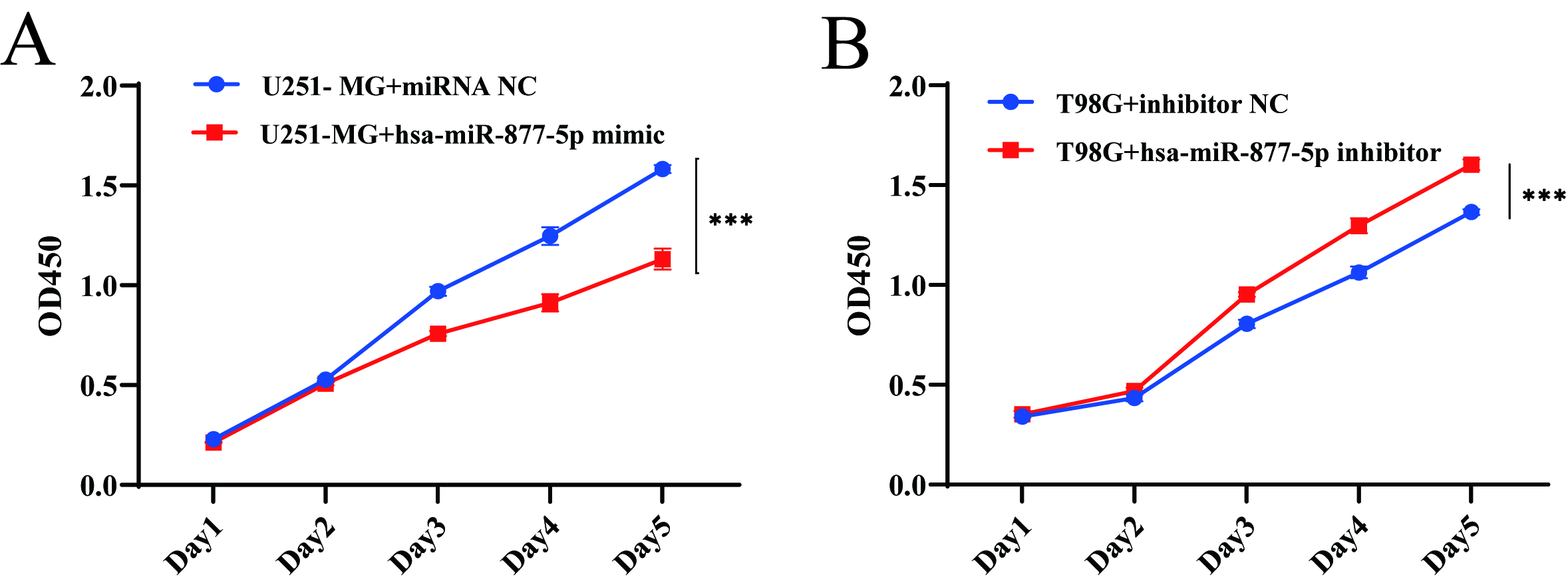

Supplement: Supplementary Figure 1 — CCK-8 assays were performed after cells were cotransfected with different vectors. (A) U251-MG cells co-transfected with circPIK3C2A-OE and miR-877-5p mimics was reduced compared with those in the circPIK3C2A-OE group. (B) T98G cells co-transfected with circPIK3C2A-KD and miR-877-5p inhibitor was reduced compared with those in the circPIK3C2A-KD group.Data are presented as the mean ± SEM of 3 independent experiments. Significant results are presented as *P < 0.05, **P < 0.01, ***P < 0.001, and ****P < 0.0001. [file Image_1.tif]
